# An Immunocytochemical Study of Interchromatin Granule Clusters in Early Mouse Embryos

**DOI:** 10.1155/2013/931564

**Published:** 2013-09-11

**Authors:** Irina Bogolyubova, Dmitry Bogolyubov

**Affiliations:** Laboratory of Cell Morphology, Institute of Cytology RAS, 4 Tikhoretsky Avenue, Saint Petersburg 194064, Russia

## Abstract

Interchromatin granule clusters (IGCs) are universal nuclear domains. Their molecular composition and functions were studied in detail in somatic cells. Here, we studied IGCs in the nuclei of early mouse embryos during zygotic gene activation (ZGA). We found that the size of IGCs gradually increases during realization of ZGA events. Using immunocytochemical approaches, we showed that the molecular composition of IGCs is also modified in mouse embryos. The hyperphosphorylated form of RNA polymerase II and the transcription factor TFIID have been revealed in IGCs before the end of ZGA. Association of these factors with IGCs became more noticeable during ZGA realization. Our data suggest that IGCs in early mouse embryos have some functional peculiarities connected most probably with IGC formation *de novo*. We believe that IGCs in early mouse embryos not only are storage sites of splicing factors but also may be involved in mRNA metabolism and represent the multifunctional nuclear domains.

## 1. Introduction

Interchromatin granule clusters (IGCs) are universal nuclear domains being studied intensively in a context of three-dimensional nuclear organization. IGCs are also known as nuclear speckles, splicing factor compartments, or SC35-domains, since they are highly enriched in pre-mRNA splicing factors including small nuclear (sn) RNPs and SR proteins [1–4]. One of IGC diagnostic markers is the SR-protein SC35 [5, 6].

Initially, IGCs had been discussed only as the sites of splicing machinery accumulation [7, 8], from which splicing factors are being recruited to the transcription sites [9–11]. During the last years, the concept on IGC functions has been considerably expanded. Direct participation of IGCs in transcription had been discussed on the base of detecting RNA polymerase II and some protein transcription factors in these domains [4]. Recent studies confirmed that IGCs are involved in main steps of gene expression including association of active genes in specific “neighborhoods” [12–14], and mRNA was shown to acquire the export competence in IGCs [15–17].

The bulk of studies concerning the molecular composition and possible functions of IGCs have been carried out on such experimental models as mammalian somatic tissue-cultured cells. The IGCs in early mammalian embryos remain poorly explored. There are only scarce data on IGCs at the initial stages of embryogenesis [18–20]. However, our previous observations [21] have demonstrated some distinctions of IGCs in early mouse embryos from classical speckles described in somatic cells.

The nucleus of early mammalian embryos is a very interesting model for nuclear domain studying. After fertilization, mammalian embryo nuclei remain transcriptionally silent for the appointed period. Reactivation of the transcription start is accompanied by complex molecular and structural changes known as zygotic gene activation (ZGA) [22–24]. In mice, ZGA is realized in two steps, and the chronology of these events is well documented [22–24]. Thus, functional peculiarities of early embryos allow studying the nuclei under different transcriptional conditions without artificial inhibitors.

In the present study, we used mouse embryos of age (i) 20–24 h after hCG, that is, before ZGA, (ii) 27-28 h and 32 h after hCG, that is, at the initial step of ZGA, and (iii) 46–48 h and 55 h after hCG, that is, after realization of ZGA events. We focused on IGCs at different stages of ZGA. We measured IGC size and also studied possible localization of some components of transcriptional machinery (hyperphosphorylated form of RNA polymerase II and TFIID) in IGCs.

## 2. Materials and Methods

### 2.1. Embryo Collection

Inbred BALB/c mice obtained from the animal nursery “Rappolovo” of the Russian Academy of Medical Sciences were used. Females were induced to ovulate by intraperitoneal injections of 5–10 IU of pregnant mare serum gonadotropin (Folligon, Intervet) followed 44–48 h later by 5–10 IU of human chorionic gonadotropin (hCG) (Chorulon, Intervet). The age of embryos was counted from the time of hCG injection.

The embryos were flushed from oviducts using F10 medium with HEPES buffer (Sigma, Saint Louis, MO). M3 medium [25] with BSA (4 mg/mL) and EDTA (10.8 *μ*M/mL) [26] was used for incubation of embryos in 5% CO_2_ environment at 37°C.

All experiments performed in this study were conducted in accordance with the national rules of the laboratory procedure with the use of experimental animals confirmed by the Ministry of Public Health, Order 755.

### 2.2. Antibodies

The following primary antibodies were used: mouse monoclonal antibody revealing the non-snRNP splicing factor SC35 (2.5 *μ*g/mL) [5], rabbit polyclonal antibody directed against the hyperphosphorylated C-terminal domain of RNA polymerase II, dilution 1 : 500 [26], and rabbit polyclonal antibody SI-1 raised against full length TFIID of human origin, dilution 1 : 100 (Santa Cruz Biotechnology, Inc., Cat. no. sc-273).

### 2.3. Electron Microscopy

For routine electron microscopy, the embryos were fixed in 1% glutaraldehyde (Polyscience Inc., Warrington, PA) in 0.05 M cacodylate buffer, pH 7.4 at 4°C overnight, and then postfixed in 1% OsO_4_ in the same buffer for 1 h. After dehydration in an ascending series of ethanol, the specimens were embedded in Spurr (Electron Microscopy Sciences, Washington, PA), and the resin was polymerized for 8 h at 70°C. Ultrathin sections were prepared with a Reichert Jung ultracut microtome, mounted on nickel grids, contrasted with uranyl acetate and lead citrate, and examined in a Libra 120 electron microscope at 80 kV.

### 2.4. Immunofluorescent/Confocal Microscopy

Embryos were fixed for 1.0 h in 4% formaldehyde freshly prepared from paraformaldehyde and 0.5% glutaraldehyde in PBS, then postfixed overnight in 2% formaldehyde at 4°C. The specimens were washed in PBS, permeabilized for 10 min with 0.5% Triton X-100 in PBS, and treated for 10 min with 10% fetal serum (Gibco, New York, USA) in PBS to prevent nonspecific antibody binding. The incubation in a mixture of first antibodies was carried out overnight in a moist chamber at 4°C. After rinsing in PBS, the preparations were incubated with fluorochrome-conjugated secondary antibodies for 1.5 h at room temperature. Secondary antibodies were FITC- or Alexa 568-conjugated goat anti-mouse or goat anti-rabbit IgG (Molecular Probes) diluted 1 : 200. After rinsing in PBS, the preparations were mounted in Vectashield (Vector Laboratories, USA).

The samples were examined with a Leica TSC SL confocal laser scanning microscope (Heidelberg, Germany) equipped with Argon (488 nm) and Helium-Neon (543 nm) lasers. Confocal images were taken with a ×63 (NA 1.32) objective. Merged images were obtained using Leica Confocal Software. Contrast and relative intensities of images were adjusted with Adobe Photoshop.

Speckle size was analyzed in computer confocal images. Five speckles were measured in three optical sections from each series, and mean average was calculated. Not less than 15 embryos were analyzed for each age group; that is, more than 200 speckles were measured for each stage studied. The smallest size of speckles that characterizes embryos of 20 h age after hCG injection was taken as a unit. Hence, the relative size of speckles was counted. The results were statistically processed using Microsoft Excel.

## 3. Results

**Figure 1:**
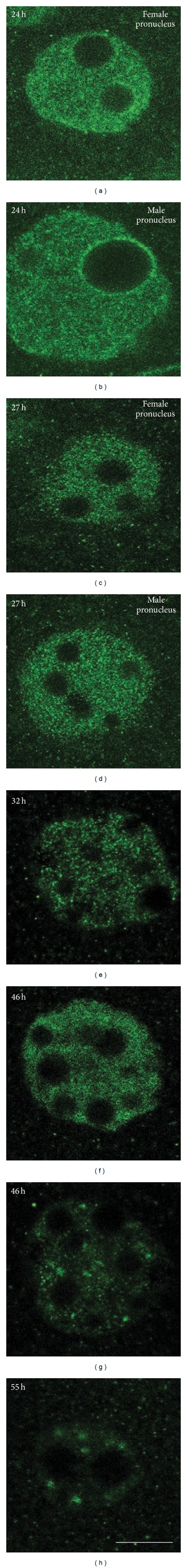
Speckles in mouse embryo nuclei at different stages of embryogenesis after immunolabeling with anti-SC35 antibody. Discrete speckles are revealed on the background of diffuse fluorescence of the nucleoplasm at all studied stages. However, the speckles in mouse embryo nuclei, especially at earlier stages, have much smaller size as compared with somatic speckles ((a)–(f)). Only in some late 2-cell embryo (g) and in 4-cell embryos (h) IGC size is similar to the size of IGCs in somatic cells. Unstained roundish areas in the nuclei correspond to the nucleolar precursor bodies (NPBs). Bar is 10 *μ*m.

Similar patterns of nuclear fluorescence were observed in embryos of all studied ages when anti-SC35 antibody has been applied (Figures 1(a)–1(h)). Prominent roundish unstained areas that are always observed in embryo nuclei correspond to nucleolar precursor bodies (NPBs), the specific nucleolar structures of the initial stages of mammal embryogenesis. Bright discrete speckles were revealed on the background of diffuse fluorescence of the nucleoplasm. The pattern of anti-SC35 staining of mouse embryos resembles well-known pattern of SC35 distribution in somatic cell nuclei [2–6]. However, speckles in mouse embryo nuclei had much smaller size compared with the size of somatic speckles that is ranged from one to several micrometers [3, 4]. Only at the late 2-cell stage and at the 4-cell stage after ZGA end (Figures 1(g) and 1(h)), IGC size in some mouse embryos was similar to the size of somatic speckles. In more than a half of 2-cell embryos, speckle size was ranged from 0.5 to 0.7 micrometers (Figure 1(f)). About 30% of 2-cell embryos displayed the size of speckles of about 1 micrometer (Figure 1(g)). In the majority of 4-cell embryos, speckles had similar size (about 1 micrometer) (Figure 1(h)), and only in 15%–20% of 4-cell embryos, speckles were smaller in size (about 0.6 micrometer).

**Figure 2:**
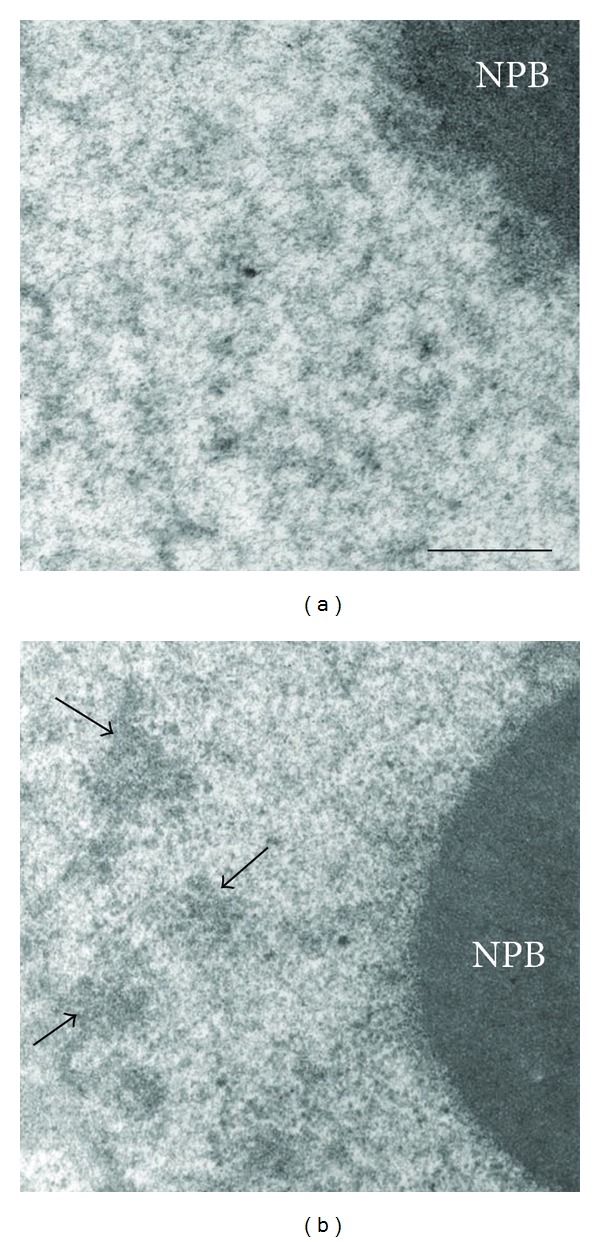
Ultrastructure of mouse embryo nuclei in early 1-cell embryo (a) and in late 2-cell embryo (b). At the ultrastructural level, typical IGCs (arrows) are clearly revealed in transcriptional active 2-cell embryos (b) but not in 1-cell embryos (a). Nucleolar precursor body (NPB). Bar is 0.5 *μ*m.

**Figure 3:**
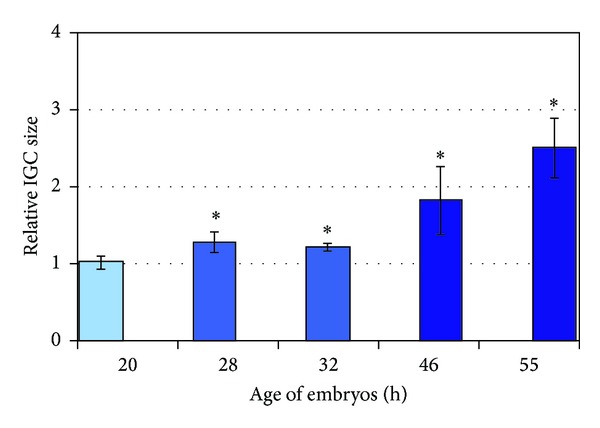
Relative sizes of IGCs in embryos, distinguished by the age. Results are presented as mean ± SEM with the level of significance *P* < 0.05. Digital images of embryo nuclei stained by antibody against SC35, a marker of speckles/IGCs, were used to measure the size of speckles. The average size of speckles at the stage of early zygote (20 h) was taken as a unit. The intensity of the column color conventionally reflects differences in transcriptional activity of embryo nuclei. The values of the relative size of speckles after the beginning of ZGA differ significantly from the stage of early zygote (20 h) at all stages studied (28 h, 32 h, 46 h, and 55 h), as indicated by asterisks. Not less than 15 embryos were analyzed for each age group; that is, more than 200 speckles were measured for each stage studied.

At the ultrastructural level, typical IGCs were clearly revealed in transcriptional active 2-cell embryos but not in 1-cell embryos (Figures 2(a) and 2(b)). Statistical analysis has confirmed that average size of IGCs is being reliably increased during realization of ZGA events (Figure 3).

**Figure 4:**
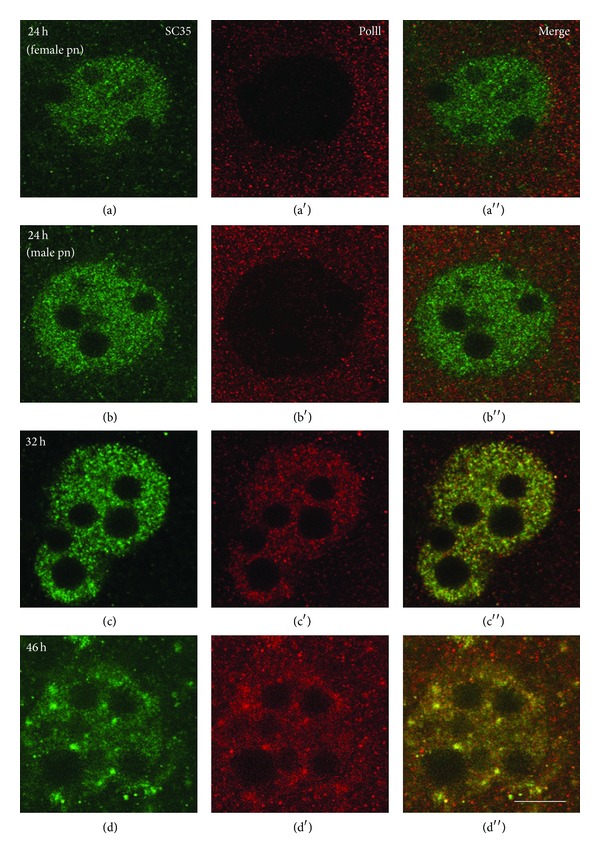
Double immunolocalization of SC35 (column (a)) and hyperphosphorylated form of RNA polymerase II (column (a′)) in mouse embryos. The hyperphosphorylated form of RNA polymerase II is not revealed in the pronuclei of 1-cell embryos (lines (a), (b)). The appropriate labeling begins to be detected only at the early 2-cell stage (line (c)). However, association of RNA polymerase II with SC35 domains (speckles) is observed already at this stage and increased when ZGA finishes (line (d)). Bar is 10 *μ*m.

**Figure 5:**
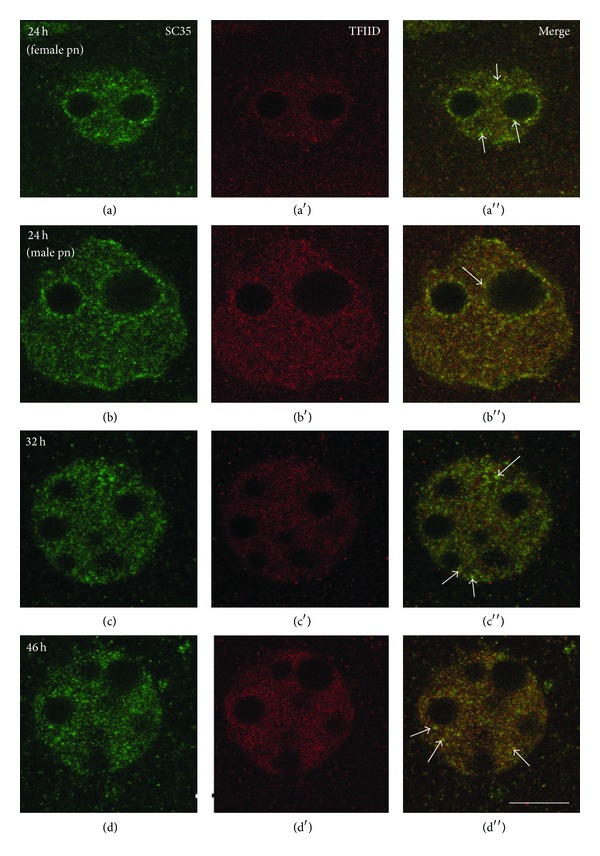
Double immunolocalization of SC35 (column (a)) and transcription factor TFIID (column (a′)) in mouse embryos. TFIID is revealed in the nuclei at all studied stages ((a′)–(d′)). Colocalization of SC35 and TFIID (arrows) is intensified during ZGA. Bar is 10 *μ*m.

The hyperphosphorylated form of RNA polymerase II was not revealed in the pronuclei of 1-cell embryos (Figures 4(a) and 4(b)). The appropriate labeling begins to be detected only at the early 2-cell stage (Figure 4(c)). However, association of RNA polymerase II with SC35 domains (speckles) was observed already at this stage and increased when ZGA has finished (Figure 4(d)). On the contrary, the transcription factor TFIID was revealed in association with nuclear speckles at all studied stages (Figures 5(a)–5(d)). It is noticeable that both TFIID and SC35 were clearly detected near the periphery of NPB at the earliest stages of cleavage (Figures 5(a) and 5(b)).

## 4. Discussion

The timing of ZGA in mouse embryos has been described in detail (for a review, see [24]). ZGA in mice is realized in two main steps. The weak transcriptional activity is revealed at the middle 1-cell stage (the so-called minor ZGA), whereas full transcription reactivation occurs at the middle 2-cell stage (the so-called major ZGA). Thus, the embryo ages which we have chosen for the present study allow comparing the morphology and molecular composition of IGCs in nuclei with different transcriptional status.

Transcriptionally active late 2-cell and 4-cell mouse embryos are characterized by larger IGCs as compared with 1-cell and early 2-cell embryos before ZGA ending. This observation makes the IGCs of mouse embryos somewhat different in comparison with typical IGCs of somatic cells. Transcriptionally silent nuclei of somatic cells including the cells experimentally treated with drugs to inhibit transcription contain large IGCs that accumulate mRNA metabolism machinery [27–30]. Thus, correlation between the size of IGCs and transcriptional activity differs in early mammalian embryos and somatic cells. However, experimental transcription inhibiting in late 2-cell mouse embryos provokes the appearance of extremely large IGCs/speckles [21, 31].

The presence of RNA polymerase II and basal transcription factor TFIID in IGCs of mouse embryos is in agreement with the results of studies carried out on somatic cells. Some authors have reported that IGCs contain the hyperphosphorylated form of RNA polymerase II [32, 33]. These data have been confirmed by IGC proteome analysis [34, 35].

We found that RNA polymerase II and TFIID appear in IGCs/speckles at different stages of mouse embryogenesis. However, TFIID is revealed in speckles even in transcriptionally silent nuclei. The localization of TFIID and SC35 in association with the periphery of NPB suggests that the functions of NPBs might be wider than it is assumed. The NPBs are known as provisional structures, some of which are able to transform into functionally competent active nucleoli (for review, see [36]). It cannot be excluded that NPBs may take part in the formation of other nuclear domains during early mammalian development. At least, there are data on the association of Cajal body precursors in the vicinity of NPBs in mammalian embryos [37].

Hence, our present data and observations that have been reported previously [31] suggest that IGCs in early mouse embryos not only are storage sites for splicing factors, but also might be involved in mRNA metabolism, representing multifunctional nuclear domains. In particular, some authors have suggested that IGCs represent the hubs of specific nuclear activities, coordinating the processes of gene expression [38]. However, in comparison with typical speckles/IGCs of somatic cells, these nuclear domains in early mouse embryos have some functional peculiarities that emphasize the uniqueness of early mammalian embryos as experimental models to explore nuclear structure and metabolism.
